# An Improved Real-Coded Genetic Algorithm Using the Heuristical Normal Distribution and Direction-Based Crossover

**DOI:** 10.1155/2019/4243853

**Published:** 2019-11-14

**Authors:** Jiquan Wang, Mingxin Zhang, Okan K. Ersoy, Kexin Sun, Yusheng Bi

**Affiliations:** ^1^College of Engineering, Northeast Agricultural University, Harbin, Heilongjiang 150030, China; ^2^School of Electrical and Computer Engineering, Purdue University, West Lafayette, IN 47907-1285, USA

## Abstract

A multi-offspring improved real-coded genetic algorithm (MOIRCGA) using the heuristical normal distribution and direction-based crossover (HNDDBX) is proposed to solve constrained optimization problems. Firstly, a HNDDBX operator is proposed. It guarantees the cross-generated offsprings are located near the better individuals in the population. In this way, the HNDDBX operator ensures that there is a great chance of generating better offsprings. Secondly, as iterations increase, the same individuals are likely to appear in the population. Therefore, it is possible that the two parents of participation crossover are the same. Under these circumstances, the crossover operation does not generate new individuals, and therefore does not work. To avoid this problem, the substitution operation is added after the crossover so that there is no duplication of the same individuals in the population. This improves the computational efficiency of MOIRCGA by leading it to quickly converge to the global optimal solution. Finally, aiming at the shortcoming of a single mutation operator which cannot simultaneously take into account local search and global search, a Combinational Mutation method is proposed with both local search and global search. The experimental results with sixteen examples show that the multi-offspring improved real-coded genetic algorithm (MOIRCGA) has fast convergence speed. As an example, the optimization model of the cantilevered beam structure is formulated, and the proposed MOIRCGA is compared to the RCGA in optimizing the parameters of the cantilevered beam structure. The optimization results show that the function value obtained with the proposed MOIRCGA is superior to that of RCGA.

## 1. Introduction

The genetic algorithm (GA) was proposed by Professor Holland and his students at the University of Michigan at the end of the 1960s and in the early 1970s [1–4]. In 1975, De Hong first proposed the evolutionary strategy (ES) of elitism preservation in his doctoral thesis. Later, others further studied it and proposed several elitism preservations and ES of replacing copying with selection [5–8]. At present, the GA is basically calculated according to these evolutionary strategies. In 1989, Goldberg [9] made a comprehensive and systematic summary and discussion of the GA and laid the foundation of the modern GA.

Initial GA uses binary coding. Because it can only map to discrete values in the search space and has Hamming distance, the accuracy of solution is not high [10]. In addition, binary coding needs to be encoded and decoded frequently, thus increasing the calculation time, with potential conversion error. Since the accuracy of the solution is controlled by the encoding length, for high accuracy, binary coding may need too long codes, resulting in excessive computing and memory space as well as reduced computational speed. In 1989, Lucasius et al. first proposed real-coded genetic algorithms (RCGAs) [11, 12]. RCGA has many attractive properties such as high precision, no coding and decoding required, effective large space search, simple computing, fast convergence, and not easy to fall into a local extreme value [13, 14].

GA has been widely applied in areas such as function optimization, automatic control, machine learning, combinatorial optimization, production scheduling problems, image processing, self-adaptation control, planning and design, industrial engineering, intelligent manufacturing systems, bioengineering, system engineering, artificial intelligence, intelligent machine system, artificial life, text information filtering, and cooperative multi-objective evaluation [15–22]. It is especially suitable to deal with complex and nonlinear continuous optimization problems that cannot be solved by traditional search methods [6].

During the last few decades, the RCGA has attracted a lot of attention because of its unique and excellent performance. Many scholars have made in-depth research on RCGA and achieved excellent research results [23–31]. Because crossover operators and mutation operators have a great influence on the performance of GA, many scholars have focused their attention on the improvement of crossover operators and mutation operators. In 1992, Wright [32] proposed a heuristic crossover (HX) operator, which is used to solve constrained and unconstrained optimization problems and achieved good results. The two offsprings generated by the HX operator are located on the straight line connecting the two parents, and are in the vicinity of the parent individuals with better fitness value. In 1993, Eshelman et al. [23] used the concept of interval schemata to develop a blend crossover operator (BLX-*α*). When the difference between the parents is small, the offsprings are similar to the parents, but if the difference between the parents is large, the generation of the offspring is similar to a random search. The parameter *α* is used to control the search domain, and the optimal value of *α* is suggested to be 0.5; BLX-*α* generates the offspring solution at the center from both selected parents. In 1995, Deb et al. [33] proposed a simulated binary crossover (SBX) which simulates the single-point binary crossover and generates two offsprings from two selected parents. The two offsprings generated by the SBX operator are located on the straight line connecting the two parents and are in the vicinity of the two parents. The distribution index *η* in the SBX operator controls the distance between two offsprings and two parents. If the value of *η* is large, the probability that the generated offsprings are closer to the two parents is greater; if the value of *η* is relatively small, the probability that the generated offsprings are farther away from the two parents is greater. The disadvantage of SBX is that it cannot adaptively control the size of the parameter *η* value, and therefore cannot adaptively control the distance between two offsprings generated by the crossover and the two parents. In 1997, Ono et al. [34] proposed the unimodal normal distribution crossover (UNDX) operator which used an ellipsoidal probability distribution to produce two or more offsprings from three selected parents. In this scheme, the offsprings located near the center of the first two parents are produced with a higher probability whereas those neighboring the parents are produced with a lower probability. In 2002, Deb et al. [35] developed a parent-centric crossover (PCX) operator. PCX is a self-adaptive multi-parent crossover which uses a large probability rather than the center of selected parents to generate a new solution near each parent. One parent is selected for each generated offspring and a difference vector is calculated between this parent and the N chosen parents. Besides, the PCX has difficulties to solve multimodal problems. The comparison results show that RCGA based on PCX outperforms other comparative crossover schemes in finding the global optimum for a number of test problems. In 2007, Deep et al. [36] proposed a Laplace crossover (LX) in which Laplace distribution is used as the density function to decide the location of the offspring. The two offsprings generated by the LX operator are symmetrical relative to their parental position, and the two offsprings are not necessarily located near the better among the two parents. In 2016, Chuang et al. [27] proposed a direction-based crossover (DBX) which is able to explore 2^*n*^ − 1 possible search directions. As a result, a high probability to produce better offspring individuals is assured. However, the search directions of DBX are limited. Although it may generate a crossover direction capable of guiding the chromosomes to move towards the optimal solution; this is not highly probable. Meanwhile, when the dimensionalities of the variables are small, the null vector solution is likely to be generated. Currently, the search direction is randomly generated, and the search direction is not instructive. In 2018, Wang et al. [37] developed a heuristic normal distribution crossover (HNDX) operator. It can guarantee the cross-generated offspring to locate closer to the better one among the two parents and the search direction to be very close to the optimal search direction or to be consistent with the optimal searching direction. However, HNDX does not consider whether there are better individuals in the population than the two parents of participation crossover, and if so, whether it should be considered in the crossover operator.

Similarly, several other mutation schemes are reported in the literature. In 1996, Michalewicz [38] proposed a non-uniform mutation (NUM) operator. At the beginning of the iterations, NUM's global search ability is strong; in the latter part of the iterations, NUM's local search ability is strong. The disadvantage of the NUM operator is that it is difficult to choose a maximum number of iterations that applies to all problems. In 1995, Hinterding [39] proposed a Gaussian mutation operator in which the global and local search capabilities of GA are changed by adjusting the variance of the Gaussian mutation operator. The disadvantage of Gaussian mutation is that the local search ability is stronger than the global search ability, and the algorithm is easy to fall into local optimum. Therefore, Gaussian mutation is not suitable for solving multimodal optimization problems. In 1996, Wang et al. [40] proposed a mutation operator which mutated towards the gradient direction of the objective function. However, when the objective function is not differentiable, the mutation operator cannot be performed. In 2014, Deb et al. [41] proposed a polynomial mutation (PM) operator, which is widely used in RCGA. PM operators have strong random search ability and are not easy to fall into local optimum, but the convergence speed of the algorithm is slower. In 2016, Chuang et al. [27] proposed a dynamic random mutation (DRM) operator. Here, the calculation formula of the step size contradicts its interpretation. In addition, the mutation operator needed the maximum number of iterations in advance, which is difficult to estimate.

In the improved RCGA mentioned above, two parents generate two offsprings by crossover. In the process of biological evolution, a pair of parents usually breed more than two offsprings. Such species not only survive well in the process of evolution, but also make the species evolve so as to get better species. In fact, there is no species in which the number of offsprings bred by a pair of parents is less than or equal to two. Even if such species exist, in the process of evolution, they will eventually become extinct due to diseases, lack of food, and other factors. In addition, when the number of offsprings within a population is greater than that of the parents, the population size will expand. If the survival space of the species is constant, the competition within the population will aggravate. In this case, excellent individuals with strong viability within the population are easy to survive, and individuals with lower viability are eliminated, so that the species gradually evolves to adapt to the living environment and get better species. Therefore, during the evolution of species in nature, the number of offsprings should be greater than that of the parents.

Inspired by the laws of biological evolution in nature, in 2015, Wang et al. [42] proposed a multi-offspring GA with single-point crossover and verified its effectiveness of single-point crossover multi-offspring GA by using test functions. In 2016, Wang et al. [43] developed multi‐offspring GA. In this, the theoretical basis of multi-offspring GA was given. The schema theorem proved that multi-offspring GA is better than non-multi-offspring GA. This paper is only suitable for solving TSP. In 2016, Wang et al. [44] proposed a multi-offspring GA of two-point crossover and verified its validity using test functions. The multi-offspring GA given in the literature is mainly divided into two categories: one is the multi-offspring GA for solving the TSP; the other is the multi-offspring GA with binary coding.

In summary, the two offsprings generated by the LX operator are symmetric with respect to the parental position, and the two offsprings are not necessarily located near the better individuals of the two parents. The two offsprings generated by the SBX operator are located on the straight line connecting the two parents, and are in the vicinity of the two parent individuals. The two offsprings generated by the HX operator are located on the straight line connecting the two parents, and are in the vicinity of the parent individuals with better fitness value. The offspring individuals generated by PCX operators are not close to the center of the parental individuals, but close to the parental individuals. The two offsprings generated by the BLX-*α* operator locate at the center of the two parents of participation crossover. Since the DBX operator is only able to explore 2^*n*^ − 1 possible search directions, the search directions of DBX are limited when the dimensionalities of the variables are small, and the null vector solution is likely to be generated. Currently, the search direction is randomly generated, and the search direction is not instructive. In order to overcome the shortcomings of the above crossover operator, this paper develops a heuristic normal distribution and direction-based crossover (HNDDBX) operator. HNDDBX not only considers the optimal individuals in the population, but also ensures that the offsprings generated after crossover are located near the better parent among the parents of participation crossover, not only near the better parent on the straight line connecting the two parents. In addition, a single mutation operator is difficult to take into account both global and local search abilities. For this reason, this paper develops a method of Combinational Mutation which includes three-mutation operators. Furthermore, because multi-offspring GA is superior to non-multi-offspring GA, the number of offsprings generated by the HNDDBX operator is more than those of the parents. Taking the above factors into consideration, this paper proposes a MOIRCGA, and it is compared with other GAs in the literature. The comparison results show that the performance of MOIRCGA is better than those of other GAs in the literature. Finally, MOIRCGA is applied to the optimization of the cantilever beam structure, and good results are obtained.

## 2. Penalty Function Method for Constrained Optimization Problems

The mathematic model of a constrained optimization problem can be generally expressed as follows:

(1) $$\begin{array}{c} \left\{\begin{array}{c} \min\;f\left(X\right),\\ \text{s}.\text{t}.\left\{\begin{array}{c} h_{i}\left(X\right)=0,\;i=1,2,\ldots ,p,\\ g_{j}\left(X\right)\geq 0,\;\;j=1,2,\ldots ,q, \end{array}\right. \end{array}\right. \end{array}$$

where *n* is the population size, *h*_*i*_(*X*) = 0 is the *i*-th equality constraint, *p* is the number of equality constraints, *g*_*j*_(*X*) ≥ 0 is the *j-*th inequality constraint, *q* is the number of inequality constraints, and *X*_*k*_ is a *D*-dimensional vector *X*_*k*_ = (*x*_*k*1_, *x*_*k*2_,…, *x*_*kD*_).

Equation (1) can also be expressed as

(2) $$\begin{array}{c} \left\{\begin{array}{c} \min f\left(X\right),\;X=\left[X_{1},X_{2},\ldots ,X_{k},\ldots ,X_{n}\right]\in R,\\ R=\left\{X\left|h_{i}\right.\left(X\right)=0,i=1,2,\ldots ,p;g_{j}\left(X\right)\geq 0,j=1,2,\ldots ,q\right\}. \end{array}\right. \end{array}$$

Stating *X* ^*∗*^ is the optimal solution to the constrained optimization problems means that ∀*X* ∈  *R*: *f*(*X* ^*∗*^) ≤ *f*(*X*). In addition, if *g*_*j*_(*X* ^*∗*^)=0, the constraint is referred to as the active constraint. Under this concept, all the equation constraints *h*_*i*_(*X*) = 0 (*i* = 1, 2,…, *p*) are active at *X* ^*∗*^.

The penalty function method converts a constrained optimization problem to an unconstrained optimization problem by using two penalty factors and defining the penalty function to be minimized as [45]:

(3) $$\begin{array}{c} P\left(X,M\right)=f\left(X\right)+M_{1}\overset{p}{\underset{i=1}{\sum }}{\left[h_{i}\left(X\right)\right]}^{2}+M_{2}\overset{q}{\underset{j=1}{\sum }}{\left[\min\left(0,g_{j}\left(X\right)\right)\right]}^{2}, \end{array}$$

where *M*_1_ and *M*_2_ are the penalty factors, generally chosen as positive constants that are big enough; the second and third terms on the right are the penalty terms, and *P*(*X*, *M*) is the penalty function.

In equation (3), when *X* ∈ *R,* there should be no penalty to the feasible points, thus *P*(*X*, *M*) = *f*(*X*); when *X* ∉ *R*, for the nonfeasible points, *M*_1_ and *M*_2_ should be very big. Moreover, when point *X* gets farther away from the feasible region, the penalty should be larger.

The minimum value of equation (3), that is,

(4) $$\begin{array}{c} \min\;P\left(X,M\right), \end{array}$$

is equivalent to the minimum value of equation (1).

## 3. Multi-offspring Improved Real-Coded Genetic Algorithm (MOIRCGA)

In the multi-offspring GA, the number of offsprings is an integer multiple of the number of parents. Other GAs will be called conventional GAs.

Letting *n*_0_ and *n*_1_ be the number of parents and offsprings, respectively, we get

(5) $$\begin{array}{c} n_{1}=kn_{0},\;k\in \left\{2,3,4,\ldots \right\}. \end{array}$$

In the paper, the number of offsprings generated by crossover will be chosen as 2*n*. In comparing multi‐offspring GA with conventional GA, the competition among individuals in the population in multi‐offspring GA is more intensifier. Most of the surviving individuals in the population are excellent. This causes multi-offspring GA to have faster convergence speed than conventional GA.

### 3.1. Mathematical Symbols of MOIRCGA

We define the following symbols: *X* ^0^=(*X*_1_^0^,  *X*_2_^0^,…, *X*_*i*_^0^,…, *X*_*n*_^0^) is the initial population, where *X*_*i*_^0^=(*X*_*i*1_^0^, *X*_*i*2_^0^,…, *X*_*iD*_^0^), *i* = 1, 2,…, *n*; *D* and *n* are the dimensions of the variables and the population size, respectively; *a* = [*a*_1_, *a*_2_,…, *a*_*D*_] and *b* = [*b*_1_, *b*_2_,…, *b*_*D*_] are lower and upper bounds of the variables *X*_*i*_^0^. In the *t*-th iteration, *X*_*i*_(*t*) is also referred to as a chromosome with *x*_*i*1_(*t*), *x*_*i*2_(*t*),…, *x*_*i* *D*_(*t*) known as genes. We also define *P*_*c*_ and *P*_*m*_ as the crossover mutation probabilities, respectively, and *m* is the number of preserved elitism individuals.

### 3.2. Initialization of Population

Before the population is initialized, the upper and lower bounds of the variables are determined. Hereafter, the variables are randomly generated uniformly within the interval [*a*, *b*]. Therefore, the *i-*th individual *X*_*i*_^0^ in the initial population is initialized as

(6) $$\begin{array}{c} X_{i}^{0}=a+\left(b-a\right)*\text{rand}\left(1,D\right),\;i=1,2,\ldots ,n, \end{array}$$

where (*b* − *a*).*∗*rand(1, *D*) is the product of multiplying (*b* − *a*) with the element at the same position of rand(1, *D*), and rand(1, *D*) represents *D* uniformly distributed random numbers in [0, 1].

### 3.3. ES of MOIRCGA

In the population evolution process of MOIRCGA, the population size is assumed to be constant. The ES of MOIRCGA is as follows:

1. Generate the initial population of size *n*, and sort all individuals in descending order according to their objective function values.
2. Select *m* elitist individuals with best ranking among *n* parents. Use the method of sorting grouping selection (SGS) to select the parents of participation crossover as detailed in Section 3.4.
3. Let the crossover probability *P*_*c*_ equal 1 and *n* parents generate 2*n* offsprings by crossover as detailed in Section 3.5.
4. Generate a new population consisting of 2*n* offsprings and *m* elitist individuals chosen in step 3.
5. Sort the individuals of the new population in descending order according to their objective function values.
6. Select *m* elitist individuals with the best ranking in the population.
7. Using the mutation operator, modify the 2*n* offsprings generated by crossover. This is discussed in Section 3.6.
8. Regenerate the new population consisting of the 2*nP*_*m*_ mutated offsprings, 2*n*(1 − *P*_*m*_) unmutated offsprings, and *m* elitist individuals chosen in step 6.
9. Sort the individuals of the regenerated new population in descending order according to their objective function values.
10. If the iterative termination condition is met, output the optimal solution and the optimal value. Otherwise, go to the next step.
11. Select *n* individuals with best ranking within the last population. Go to step 2 to start the next iteration.

**Figure 1:**
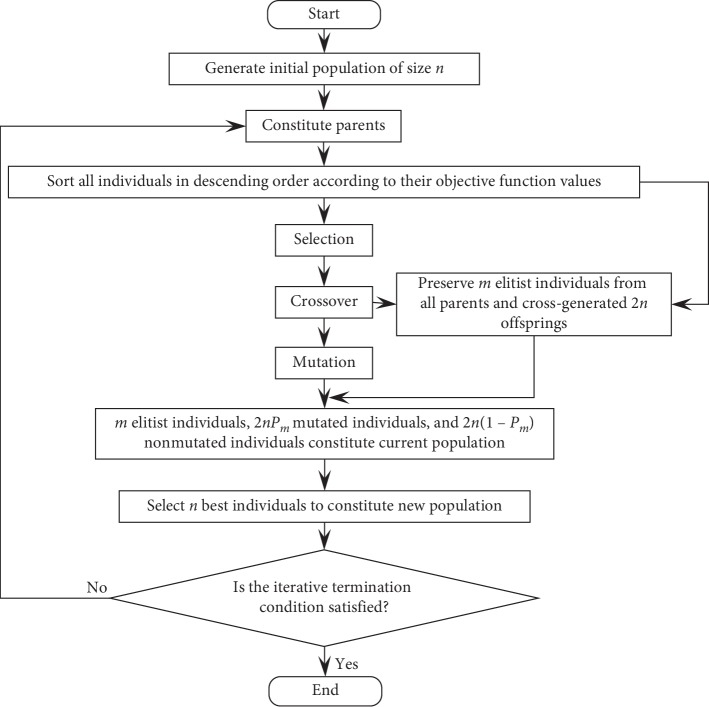
The flow diagram of MOIRCGA.

The ES flow diagram for MOIRCGA is shown in Figure 1.

In the ES above, because the crossover probability is 1, the number of new individuals generated by crossover increases, and thus the possibility of generating excellent individuals increases. In addition, the number of new individuals generated by mutation is 2*nP*_*m*_, which is twice the number of new individuals generated by conventional GA mutation. Therefore, the possibility of obtaining excellent individuals and jumping out of local optimum is increased. This helps to improve the convergence speed of MOIRCGA.

### 3.4. Sorting Grouping Selection (SGS)

We assume the following: (1) the maximum of the objective function is sought, (2) the population size *n* is an even number, and (3) the individuals in the population are sorted with respect to their objective values *P*(*X*, *M*) in descending order. If the minimum of the objective function is sought, the objective function *P*(*X*, *M*) can be changed to the maximum value by max*P*(*X*, *M*) = −min[−*P*(*X*, *M*)]. The population before sorting is *X*(*t*) = (*X*_1_(*t*), *X*_2_(*t*),…, (*X*_*n*_(*t*)), and after sorting in descending order becomes *X*′(*t*) = (*X*′_1_(*t*), *X*′_2_(*t*),…, (*X*′_*n*_(*t*)), where *P*((*X*′_1_(*t*), *M*) ≥ *P*((*X*′_2_(*t*), *M*) ≥⋯≥ *P*((*X*′_*n*_(*t*), *M*), *t* being the number of iterations.

The individuals in the population are divided into two groups according to their objective function values. Group 1 includes the better *n*/2 individuals, that is, *X*′_1_(*t*), *X*′_2_(*t*),…, *X*′_*n*/2_(*t*), and Group 2 includes the other *n*/2 individuals, that is, *X*′_*n*/2+1_(*t*), *X*′_*n*/2+2_(*t*),…, *X*′_*n*_(*t*). The 1^st^ individual in Group 1 is matched with the 1^st^ individual in Group 2, the 2^nd^ individual in Group 1 is matched with the 2^nd^ individual in Group 2, and so on. In this way, we obtain 0.5*n* pairs of individuals of participation crossover. This selection method is called SGS. The advantage of the SGS method is that the selection operation is completed according to the objective function value of each individual, and it is not necessary to calculate the fitness value of each individual. This causes selection operation speed to be fast.

### 3.5. Heuristic Normal Distribution and Direction-Based Crossover (HNDDBX)

If the optimization problem is to solve for the maximum value of the objective function, the optimal solution is likely to be near the individual with a bigger objective function value [46]. Hence, the better individuals in the population are more likely to approach the optimal solution. Inspired by this, this paper develops a heuristic normal distribution and direction-based crossover (HNDDBX) operator.

**Figure 2:**
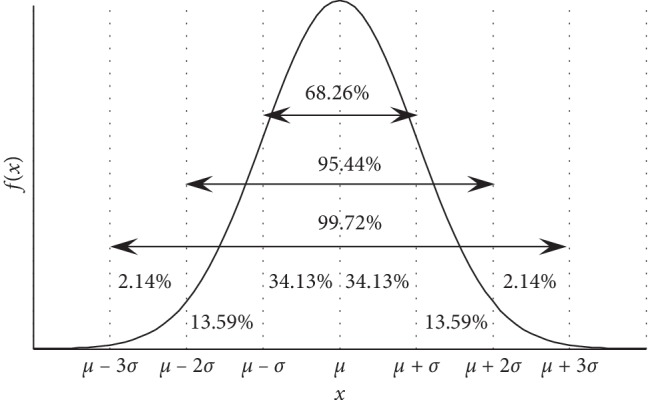
Density function of the normal distribution.

The key to HNDDBX is how to make the crossover generated offspring be in the vicinity of the better individuals in the population. Since a random number generated by the normal distribution has a higher probability of being located near its mean *μ*, if a better individual in the population is used as being the mean *μ* of the normal distribution, then it is guaranteed that its offspring according to normal distribution is in the vicinity of the better individual. Therefore, a normal distribution can be used to generate offsprings. The normal distribution is denoted by *N*(*μ*, *σ* ^2^), where *μ* is the mean, and *σ* ^2^ is the variance. The density function of the normal distribution is shown in Figure 2.

It can be seen from Figure 2 that the probability of a random number *X* generated by *N*(*μ*, *σ* ^2^) in the interval (*μ* − *σ*, *μ* + *σ*) is 68.26%, the probability of *X* in the interval (*μ* − 2*σ*, *μ* + 2*σ*) is 95.44%, and the probability of *X* in the interval (*μ* − 3*σ*, *μ* + 3*σ*) is 99.72%. It can be seen that the probability that *X* falls outside the interval (*μ* − 3*σ*, *μ* + 3*σ*) is less than 0.3%. Thus, the interval (*μ* − 3*σ*, *μ* + 3*σ*) can be regarded as the actual possible interval of the random variable *X*, which is called the 3*σ* principle of the normal distribution. Based on the above analysis, as long as we control the size of *σ* value, we can basically guarantee that the random number *X* generated by *N*(*μ*, *σ* ^2^) is near the mean *μ*. If the mean *μ* of the normal distribution is equal to the better individual *X* within the parents of participation crossover, it can be ensured that the individuals generated by the normal distribution are located near the better individual *X*.

In order to explain the HNDDBX operator, we assume that the population size *n* is even. After sorting in descending order according to objective function values, the population is *X*′ = (*X*′_1_, *X*′_2_,…, *X*′_*n*_). At the *t*-th iteration, the optimal individual in the population is *X*′_1_. This paper uses the SGS method to select individuals of participation crossover. Let the paired parents of participation crossover be *X*′_*i*_ (*i* = 1, 2,…, *n*/2) and *X*′_*j*_ (*j* = *n*/2 + 1, *n*/2 + 2,…, *n*). Assuming that *X*′_*i*_ is superior to *X*′_*j*_, the offsprings are generated by

(7) $$\begin{array}{c} \left\{\begin{array}{c} Y_{i}=N\left(\bar{X},\varepsilon +{\left[\frac{X_{i}^{\prime }-X_{j}^{\prime }}{12}\right]}^{2}\right),\\ Y_{j}=N\left(X_{1}^{\prime },\varepsilon +{\left[\frac{X_{1}^{\prime }-\bar{X}}{12}\right]}^{2}\right),\\ Y_{k}=X_{1}^{\prime }+R_{1}*\left(X_{i}^{\prime }-X_{j}^{\prime }\right),\\ Y_{l}=\bar{X}+R_{2}*\left(X_{1}^{\prime }-\bar{X}\right),\\ \bar{X}=\frac{1}{3}\left[{\bar{X}}_{1}+X_{1}^{\prime }+X_{i}^{\prime }\right],\\ {\bar{X}}_{1}=\frac{X_{1}^{\prime }+X_{2}^{\prime }+\cdots +X_{0.5n}^{\prime }}{0.5n},\\ R_{1}=\left(r_{11},r_{12},\ldots ,r_{1D}\right),\\ R_{2}=\left(r_{21},r_{22},\ldots ,r_{2D}\right),\\ i=1,2,\ldots ,\frac{n}{2};\;j=\frac{n}{2}+1,\;\frac{n}{2}+2,\ldots ,n;\\ k=n+1,n+2,\ldots ,\frac{3n}{2};\;l=\frac{3n}{2}+1,\frac{3n}{2}+2,\ldots ,2n, \end{array}\right. \end{array}$$

where *D* is the dimension of the variables, *Y*_*i*_ (*i* = 1, 2,…, 2*n*) are the offsprings generated by the crossovers, *r*_11_, *r*_12_,…, *r*_1*D*_ and *r*_21_, *r*_22_,…, *r*_2*D*_ are uniformly distributed random numbers in [0, 1], *ε* is a small positive number to avoid that the variance of the normal distribution is 0. *∗* means that the elements at the same position in two matrices or vectors are multiplied.

**Figure 3:**
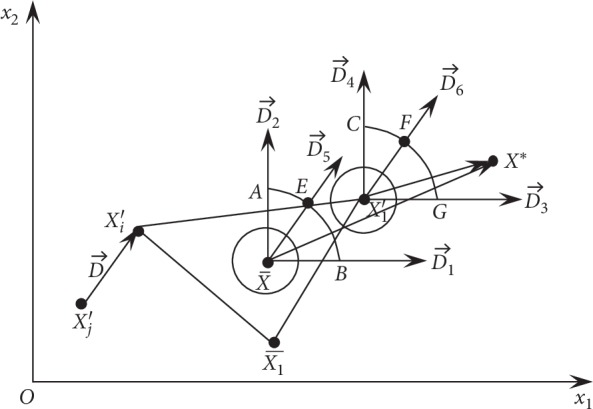
Schematic diagram of HNDDBX.

To illustrate the principle of HNDDBX, let the variable dimension *D* be 2. At the *t*-th iteration, let the two parent individuals of participation crossover be *X*′_*i*_ and *X*′_*j*_, respectively. If *X*′_*i*_ is superior to *X*′_*j*_, *X*′_1_ is the best individual in the population, and *X* ^*∗*^ is the optimal solution to the problem to be solved. The principle of HNDDBX is shown in Figure 3.

In Figure 3, $A\bar{X}=B\bar{X}=X_{1}^{\prime }C=X_{1}^{\prime }G=E\bar{X}=FX_{1}^{\prime }=\left|X_{i}^{\prime }-X_{j}^{\prime }\right|$ and $\overset{⟶}{D}={\overset{⟶}{D}}_{5}={\overset{⟶}{D}}_{6}=X_{i}^{\prime }-X_{j}^{\prime }$. Equation (7) shows that ${\bar{X}}_{1}$ is the center of the better 0.5*∗n* individuals in the population, *X*_1_′ is the best individual in the population, *X*_*i*_′ is the better one among the two parent individuals of participation crossover, and $\bar{X}$ is the center of ${\bar{X}}_{1}$, *X*_1_′, and *X*_*i*_′. So, in most cases, $\bar{X}$ is superior to *X*_*i*_′. Offsprings *Y*_*i*_ (*i* = 1, 2,…, *n*/2) are randomly generated according to $N\left(\bar{X},\varepsilon +{\left(\left(X_{i}^{\prime }-X_{j}^{\prime }\right)/12\right)}^{2}\right)$, with its mean equal to the mean of $\bar{X}$ and its variance equal to *ε*+((*X*_*i*_′ − *X*_*j*_′)/12)^2^. In addition, it can be seen from Figure 3 that the random number generated according to $N\left(\bar{X},\varepsilon +{\left(\left(X_{i}^{\prime }-X_{j}^{\prime }\right)/12\right)}^{2}\right)$ has high probability of being in the vicinity of the mean $\bar{X}$ of the normal distribution. According to the above analysis, the two parent individuals *X*_*i*_′ and *X*_*j*_′ generate offsprings *Y*_*i*_, which have a great possibility to be located within a circle with $\bar{X}$ as its center and $\bar{X}+3\left(\varepsilon +{\left(\left(X_{i}^{\prime }-X_{j}^{\prime }\right)/12\right)}^{2}\right)$ as its radius, as shown in Figure 3. When the dimension of the variables is greater than 2, *Y*_*i*_ has a great possibility to be located within a sphere with $\bar{X}$ as its center and $\bar{X}+3\left(\varepsilon +{\left(\left(X_{i}^{\prime }-X_{j}^{\prime }\right)/12\right)}^{2}\right)$ as its radius. Based on the above analysis, as long as we control the size of variance *ε*+((*X*_*i*_′ − *X*_*j*_′)/12)^2^, the HNDDBX operator can basically guarantee that the offsprings generated by crossover are near the mean $\bar{X}$. Similarly, the offspring individual *Y*_*j*_ (*j* = *n*/2 + 1, *n*/2 + 2,…, *n*) generated by crossover according to equation (7) is near the optimal individual *X*_1_′ in the population. Because *X*_1_′ is the best individual in the population, the offsprings generated by crossover with the HNDDBX operator are expected to be better than those of the crossover operator in [31, 37].

It can be seen from Figure 3 that *X*_*i*_′ − *X*_*j*_′ is the search direction for *Y*_*k*_ (*k* = *n* + 1, *n* + 2,…, 1.5*n*). When *r*_12_ = 0, since *r*_11_ is a random number uniformly distributed in [0, 1], and the search direction is ${\overset{⟶}{D}}_{3}$, *Y*_*k*_ is uniformly distributed on the line segment *X*_1_′*G*. When *r*_11_ = 0, because *r*_12_ is a random number uniformly distributed in [0, 1] and the search direction is ${\overset{⟶}{D}}_{4}$, *Y*_*k*_ is uniformly distributed on the line segment *X*_1_′*C*. When *r*_11_ = *r*_12_, since *r*_11_ and *r*_12_ are random numbers uniformly distributed in [0, 1] and the search direction is ${\overset{⟶}{D}}_{6}$, *Y*_*k*_ is uniformly distributed on the line segment *X*_1_′*F*. When *r*_11_ and *r*_12_ are random numbers uniformly distributed in [0, 1], the search directions are any directions between ${\overset{⟶}{D}}_{3}$ and ${\overset{⟶}{D}}_{4}$. At this point, there are countless search directions, and *Y*_*k*_ may be located at any point within *X*_1_′*CFG*. In addition, because *X*_1_′ is better than *X*_*i*_′, the offsprings *Y*_*k*_ generated by the HNDDBX operator have a great possibility to be superior to those of the crossover operator in [31, 37]. Thus, *Y*_*k*_ may be very close to the optimal solution *X* ^*∗*^ of the problem to be solved. When *r*_22_ = 0, since *r*_21_ is a random number uniformly distributed in [0, 1] and the search direction is ${\overset{⟶}{D}}_{1}$, *Y*_*l*_ is uniformly distributed on the line segment $\bar{X}B$. When *r*_21_ = 0, because *r*_21_ is a random number uniformly distributed in [0, 1] and the search direction is ${\overset{⟶}{D}}_{2}$, *Y*_*l*_ is uniformly distributed on the line segment $\bar{X}A$. When *r*_21_ and *r*_22_ are random numbers uniformly distributed in [0, 1], the search directions are any directions between ${\overset{⟶}{D}}_{1}$ and ${\overset{⟶}{D}}_{2}$. At this point, there are countless search directions, and *Y*_*l*_ may be located at any point within $\bar{X}AEB$; *Y*_*l*_ may be very close to the optimal solution *X* ^*∗*^ of the problem to be solved.

In summary, the offsprings generated by the HNDDBX crossover operator are better than those of the crossover operator in [31, 37]. Therefore, the HNDDBX operator can significantly improve the convergence speed of MOIRCGA.

**Figure 4:**
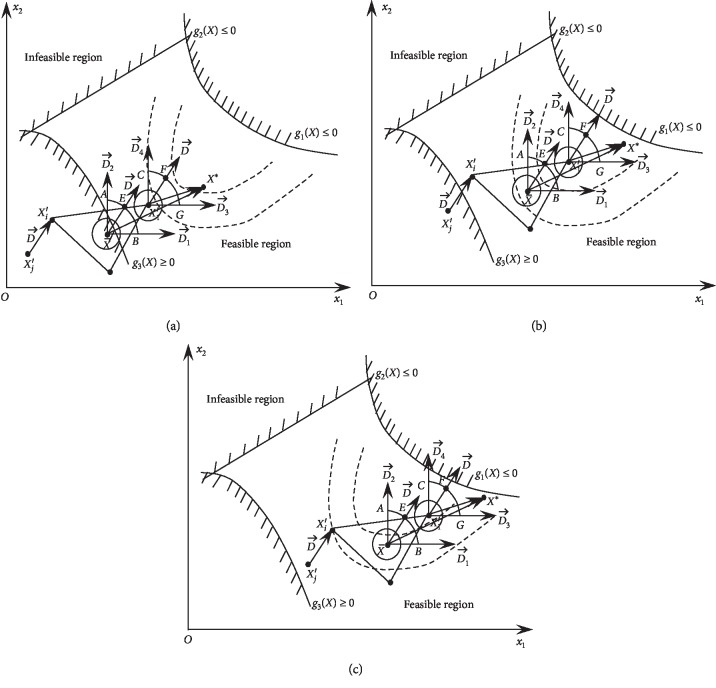
Schematic diagrams of the HNDDBX operations in a two-dimensional space under the following three cases: case (a): both parents are in the infeasible region; case (b): one parent is in the feasible region and the other one is in the infeasible region; case (c): both parents are in the feasible region.

As an example, in terms of 2*D* search space, for the spatial positions of two parent individuals *X*′_*i*_ and *X*′_*j*_ participating in crossover, there are three possible cases: (a) both parents are in the infeasible region; (b) one parent is in the feasible region while the other is not; and (c) both parents are in the feasible region. Of these three cases, the HNDDBX operation in a two-dimensional space is shown in Figure 4.

In Figure 4, the schematic of case (a) shows that the HNDDBX operator can force two parents to move from the infeasible region to the feasible region. In the case of (b), the HNDDBX operator enables the individuals in the infeasible region to move into the feasible region, and the individuals that have been in the feasible region improve their objective function values by HNDDBX. When the individuals are already in the feasible region, as in the case of (c), HNDDBX searches near the better individual and the optimal individual moves towards the global optimal solution.

### 3.6. Substitution Operation

In the global optimization problem with many local optima, when the MOIRCGA finds a region with an extreme value (whether it is a local extremum or a global extremum), individuals in the population constantly move closer to the region, and they may be the same or similar individuals in the population. With the increase of the iterations, the same individuals in the population will gradually increase and may even make all the individuals in the population to be the same. If there are many such individuals in the population, it is likely that the two parents of participation crossover are the same, such that the offsprings *Y*_*k*_ generated by crossover, according to equation (7) are the same as the two parents of participation crossover. Hence, the crossover operation does not work, and GA is unable to converge to the global optimal solution. In order to avoid the above phenomenon, a substitution operation is added after crossover operation of GA. The method of substitution operation is as follows: the population size after crossover is 2*n*; if there are two or more same individuals in the population, only one of those is retained, and the remaining individuals are removed. Let the population size be *n*_1_ at this time. In order to maintain the population size after the crossover as 2*n*, 2*n* − *n*_1_ individuals are randomly generated, and 2*n* − *n*_1_ same individuals are replaced by randomly generated 2*n* − *n*_1_ individuals. The following examples illustrate the substitution operation.

Suppose the population size is 10. After several iterations, the population is *X*. At this time, there are same individuals in the population. In order to improve the efficiency of crossover operation and avoid falling into local optimum, only one of the same individuals is retained; the population after removing the same individuals is *X*_1_, which has four fewer individuals than *X*. In order to keep the size of the population unchanged, 4 individuals *Y* are randomly generated. The population *X*′ after the substitution operation consists of *X*_1_ and *Y*. *X*, *X*_1_, and *X*′ are shown in Table 1.

### 3.7. Combinational Mutation

With the mutation operators given in the existing literature, some local search abilities are strong [47, 48], such as Gauss mutation operator, and some global search abilities are strong [48], such as Cauchy mutation operator. For optimization problems with less number of extreme points, a mutation operator with stronger local search ability should be adopted. For optimization problems with more number of extreme points, if a mutation operator with stronger local search ability is adopted, it is easy to converge to the local optimum; if a mutation operator with strong global search ability is adopted, and the accuracy requirement of the optimal solution for the problem to be optimized is higher, the convergence speed of the algorithm slows down. With some of the mutation operators given in the literature, there is strong global search capability at the beginning of the iterations, and with the increase of the number of iterations, local search capabilities are enhanced. However, this requires the maximum number of iterations to be given, which is difficult to do in advance. Although this kind of mutation operator is theoretically feasible, it actually has poor performance [27]. In summary, a single mutation operator is difficult to take into account both global and local search abilities. For this reason, we developed a method of combinational mutation which includes three-mutation operators. They are as follows.

The first mutation operator is a mutation operator based on the Cauchy distribution called the Cauchy mutation operator. The Cauchy mutation operator is as follows:

(8) $$\begin{array}{c} {\bar{X}}_{i}^{\prime }={\bar{X}}_{i}+{\bar{X}}_{i}*\text{Cauchy}\left(0,1\right), \end{array}$$

where Cauchy (0, 1) is the standard Cauchy distribution, ${\bar{X}}_{i}$ is the individual to be mutated, and ${\bar{X}}_{i}^{\prime }$ is the individual after the mutation.

The second one is the mutation operator based on the normal distribution called the normal mutation. The normal mutation operator is as follows:

(9) $$\begin{array}{c} {\bar{X}}_{i}^{\prime }=N\left(\mu ,\delta^{2}\right), \end{array}$$

where *μ* is the mean of the normal distribution, usually chosen as *μ* = ${\bar{X}}_{i}$, and *δ* ^2^ is the variance of the normal distribution.

*δ* ^2^ of (9) is given by

(10) $$\begin{array}{c} \delta^{2}={\left(\frac{X_{\text{best}}-{\bar{X}}_{i}}{12}\right)}^{2}, \end{array}$$

where *X*_best_ is the optimal individual in the population.

The third one is the mutation operator based on Lévy flight, called the Lévy mutation. It is as follows:

(11) $$\begin{array}{c} {\bar{X}}_{i}^{\prime }={\bar{X}}_{i}+\alpha *\text{Lévy}\left(\lambda \right), \end{array}$$

where *α* is the step size scaling factor, generally chosen as *α* = 0.01.

The calculation formula for the simulated Lévy flight path proposed by Mantegna is as follows [49]:

(12) $$\begin{array}{c} \text{Lévy}\left(\lambda \right)=\frac{\mu }{{\left|v\right|}^{1/\lambda }}, \end{array}$$

where 0 < *λ* < 2, usually chosen as *λ* = 1.5.

(13) $$\begin{array}{c} \left\{\begin{array}{c} \mu =N\left(0,\sigma_{\mu }^{2}\right),\\ v=N\left(0,\sigma_{v}^{2}\right), \end{array}\right.\\ \left\{\begin{array}{c} \sigma_{\mu }={\left\{\frac{\Gamma \left(1+\lambda \right)\sin\left(\pi \lambda /2\right)}{\Gamma \left[\left(1+\lambda \right)/2\right]\lambda 2^{\left(\lambda -1\right)/2}}\right\}}^{1/\lambda },\\ \sigma_{v}=1, \end{array}\right. \end{array}$$

where Γ is the Gamma function.

**Figure 5:**
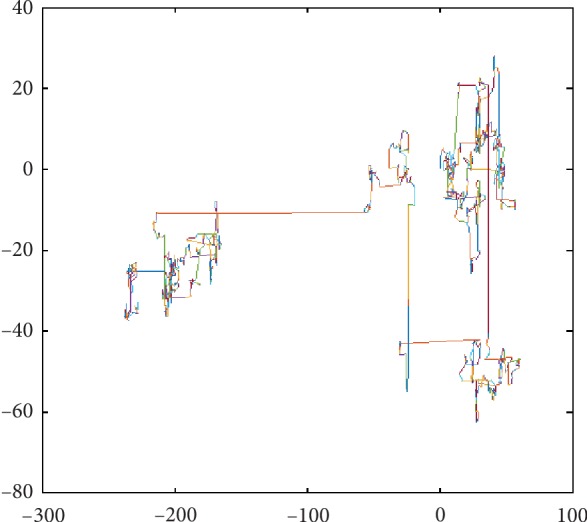
The two-dimensional space Lévy flight diagram.

The two-dimensional space Lévy flight diagram is shown in Figure 5.

As seen in Figure 5, the characteristics of Lévy flight are as follows: (1) there are a lot of small steps, that is, most of the time, it gives a local search; (2) sometimes there is a large displacement, so that individuals in the population do not search only in one place, resulting in occasional global search capability.

The specific method of combinational mutation is as follows: in the *t*-th iterations, we divide the number of iterations by 3. When the remainder is 1, the first mutation operator is used; when the remainder is 2, the second mutation operator is used; when the remainder is 0, the third mutation operator is used.

Compared with the normal mutation operator, the generated offspring of Cauchy mutation operator has high probability of being far away from the individual to be mutated, so the global search ability of Cauchy mutation operator is strong. The normal mutation operator focuses on searching for a local region near the individual to be mutated, and the local search ability is stronger, but the ability to guide the individual to jump out of the local extremum is weak, which is not conducive to global convergence. The Lévy mutation operator performs local searches in most cases but occasionally performs global searches. Therefore, the advantage of combinational mutation is that both the local search ability and the global searching ability are taken into account.

### 3.8. Pseudocode of MOIRCGA

The pseudocode of MOIRCGA is shown in Algorithm 1.

## 4. Algorithmic Testing and Analysis

RCGA proposed in [27, 31, 37] is abbreviated as IRCGA-1, IRCGA-2, and IRCGA-3, respectively. The single-point crossover multi-offspring GA proposed in [42] is abbreviated as SPXMOGA, and hybrid GA in [50] is abbreviated as HGA.

In order to verify the validity and feasibility of MOIRCGA, it is compared with IRCGA-1, IRCGA-2, IRCGA-3, SPXMOGA, and HGA.

### 4.1. Iteration Termination Condition

The iteration termination condition for GA is to be defined as

(14) $$\begin{array}{c} \left|f_{i}-f_{i}^{*}\right|\leq \varepsilon_{i},\;i=1,2,\ldots ,p, \end{array}$$

where *f*_*i*_^*∗*^ is the true global optimal value of the *i-*th test function, *f*_*i*_ is the optimal value of the *i-*th test function obtained by GA, and *ε*_*i*_ is the error for the *i-*th test function.

### 4.2. Test Functions

Sixteen well-known benchmark constrained functions were selected and are described in Appendix A [27, 31, 37].

### 4.3. Parameter Settings

In order to obtain a fair performance comparison, the parameters are set as follows.

(1) The computing precision of various test functions: *ε*_1_ = *ε*_2_ = *ε*_3_ = *ε*_4_ = *ε*_5_ = *ε*_6_ = *ε*_7_ = *ε*_8_ = *ε*_9_ = *ε*_11_ = *ε*_12_ = *ε*_13_ = *ε*_14_ = *ε*_15_ =*ε*_16_ = 10^−4^, *ε*_10_ = 10^−2^; (2) the population size *n* = 100; (3) *M*_1_ = 10^9^ and *M*_2_ = 10^7^ in the penalty function; (4) in MOIRCGA, the mutation probability *P*_*m*_ = 0.5 and the crossover probability *P*_*c*_ = 1; (5) in IRCGA-1, IRCGA-2, IRCGA-3, SPXMOGA, and HGA, the parameter settings are the same as in [27, 31, 37, 42, 50]; (6) reserve 50 elite individuals in MOIRCGA; and (7) the ES of IRCGA-1, IRCGA-2, IRCGA-3, and SPXMOGA are the same as that of in [27].

### 4.4. Comparison with the Results of Other Studies in the Literature

16 test functions in Appendix A were used as examples and each test function was run on the same computer for 1000 times. The initial population of the various algorithms is the same.

All of the algorithms in this paper were developed in *Matlab R*2018*b* programming language. IRCGA-1 [27] proposed a direction-based heuristic crossover operator which is also used in this paper. IRCGA-2 [31] gives a selection method and a direction-based heuristic crossover operator. IRCGA-3 [37] gives a heuristic normal distribution crossover (HNDX) operator. However, the HNDX does not consider the influence of the optimal individual in the population on the offsprings. SPXMOGA [42] proposes a single-point crossover multi-offspring GA based on binary coding and gives the generation method of multi-offspring and the corresponding evolutionary strategy, where the crossover operator is not heuristic. A new hybrid GSA-GA algorithm is presented in [50], and a HNDX operator is used in program. All of the above algorithms have parallelism, and regardless of whether the problem to be optimized has a derivative, it can be solved by the above algorithms. The computational results of various algorithms are shown in Table 2.

The average running time and the average number of iterations at convergence to the optimal solution were calculated as follows: when the iteration termination condition is satisfied, the numbers of iterations and time of processing at the *i-*th run are, respectively, iter(*i*) and *t*(*i*), *i* = 1, 2,…, *k*, where *k* is the number of runs used in each experiment. Then, the average running time and the average number of iterations are computed by

(15) $$\begin{array}{c} t_{\text{ave}}=\frac{1}{k}\overset{k}{\underset{i=1}{\sum }}t\left(i\right),\\ {\text{iter}}_{\text{ave}}=\frac{1}{k}\overset{k}{\underset{i=1}{\sum }}\text{iter}\left(i\right), \end{array}$$

where *t*_ave_ is the average running time and iter_ave_ is the average number of iterations.

When the average running time of ICGA-1 is *t*_1_, the average running time of MOIRCGA is *t*_2_; MOIRCGA reduces the average running time by *x*% for the *i*-th test function *f*_*i*_ in comparison to IRCGA-1. *x*% is computed by

(16) $$\begin{array}{c} t_{2}\left(1-x\%\right)=t_{1}, \end{array}$$

(17) $$\begin{array}{c} x=100\left(1-\frac{t_{1}}{t_{2}}\right). \end{array}$$

The computational method of *x*% is also the same as equations (16) and (17) for IRCGA-2, IRCGA-3, SPXMOGA, and HGA.

It is observed in Table 2 that the average running time and the average number of iterations of MOIRCGA are significantly superior to those of IRCGA-1, IRCGA-2, IRCGA-3, SPXMOGA, and HGA. MOIRCGA reduces the average running time by 86.4723%, 78.1382%, 77.1723%, 92.3419%, and 7.7381% for *f*_1_, 92.9825%, 81.4815%, 68.7500%, 94.8586%, and 51.2195% for *f*_2_, 92.5178%, 33.6842%, 47.5000%, 92.3544%, and 7.3529% for *f*_3_, 92.9012%, 36.1111%, 47.7273%, 96.2388%, and 65.9259% for *f*_4_, 76.2048%, 15.0538%, 16.8421%, 88.0030%, and 11.7318% for *f*_5_, 73.8462%, 56.4103%, 51.7730%, 74.9077%, and 88.6855% for *f*_6_, 84.8276%, 85.3982%, 84.6154%, 89.7727%, and 77.0302% for *f*_7_, 60.3208%, 55.6854%, 45.9896%, 81.7335%, and 41.9980% for *f*_8_, 93.1741%, 78.0220%, 66.3866%, 93.9668%, and 54.7170% for *f*_9_, 20.0993%, 56.8114%, 51.5437%, 62.8427%, and 15.8803% for *f*_10_, 70.5069%, 58.9744%, 53.6232%, 96.5720%, and 53.6232% for *f*_11_, 72.1264%, 70.2910%, 44.5714%, 91.5725%, and 31.4488% for *f*_12_, 91.5225%, 37.7119%, 12.5000%, 92.3676%, and 5.7692% for *f*_13_, 81.4116%, 15.8228%, 7.3171%, 87.5468%, and 7.9585% for *f*_14_, 79.3843%, 85.8515%, 78.6885%, 98.8135%, and 64.6965% for *f*_15_, and 48.7271%, 51.5362%, 46.5960%, 86.8151%, and 16.0934% for *f*_16_ in comparison to IRCGA-1, IRCGA-2, IRCGA-3, SPXMOGA, and HGA. Similarly, the number of iterations are reduced by 93.0655%, 90.8858%, 87.9022%, 99.0044%, and 63.7673% for *f*_1_, 88.8700%, 82.9963%, 64.0432%, 7.1735%, and 45.0541% for *f*_2_, 91.3308%, 76.9546%, 80.0369%, 84.7351%, and 35.6557% for *f*_3_, 84.5708%, 67.1235%, 72.3050%, 98.7247%, and 58.4379% for *f*_4_, 21.6585%, 42.5447%, 45.4985%, 95.1432%, and 11.8526% for *f*_5_, 68.5968%, 75.5886%, 73.4671%, 94.0034%, and 81.0207% for *f*_6_, 45.0158%, 90.6654%, 89.8940%, 91.3991%, and 38.1776% for *f*_7_, 31.3400%, 51.7993%, 71.8694%, 92.8838%, and 18.6654% for *f*_8_, 78.6502%, 84.2126%, 79.0909%, 86.8434%, and 23.1099% for *f*_9_, 28.9076%, 72.8941%, 70.3852%, 74.0789%, and 23.3931% for *f*_10_, 73.1964%, 78.7951%, 75.3992%, 83.8478%, and 18.9170% for *f*_11_, 61.3726%, 77.4691%, 65.1300%, 84.6915%, and 9.6795% for *f*_12_, 86.5586%, 69.7727%, 28.6090%, 89.1997%, and 18.1336% for *f*_13_, 33.0088%, 52.0363%, 37.0399%, 68.3551%, and 5.7958% for *f*_14_, 26.7832%, 88.9896%, 86.2090%, 89.4509%, and 12.6428% for *f*_15_, and 19.7828%, 57.7981%, 52.5073%, 80.9492%, and 32.3387% for *f*_16_. Thus, MOIRCGA is observed to be superior to IRCGA-1, IRCGA-2, IRCGA-3, SPXMOGA, and HGA in terms of all the measures utilized.

## 5. Parameter Optimization of Cantilever Beam

**Figure 6:**
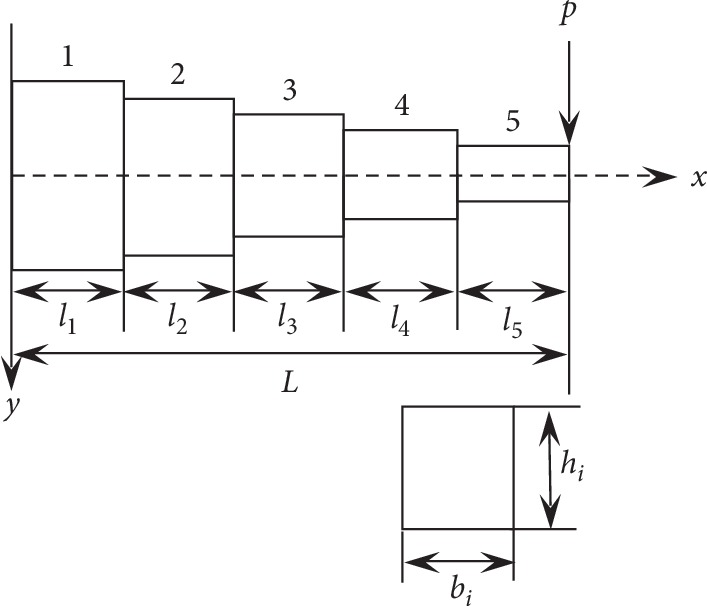
Schematic of the cantilevered beam structure with its design variables.

To further verify the validity of the MOIRCGA, the cantilever beam design problem with discrete cross-sectional area given in [50] was chosen as an application. The cantilever beam structure is shown in Figure 6.

The goal of the cantilever beam design optimization problem is to determine the optimal combination of five different cross-sectional areas to minimize the volume of the cantilever beam. The design problem has 10 variables. They are the width and height of each cross section, *h*_*i*_ and *b*_*i*_ (*i* = 1, 2,…, 5). An external force *p* = 50,000 N is applied at the free end of the cantilevered beam. The maximum allowable stress at the end of each section is *σ*_max_ = 14,000 N/cm^2^, the material elasticity modulus *E* is 200 Gap, the length of each section *l*_*i*_ (*i* = 1, 2,…, 5) is 100 cm, and the maximum allowable deflection is *y*_max_ = 2.715 cm. The height-to-width aspect ratio of each cross-section is restricted to be less than 20. Then, the mathematical model for the optimization of this problem is defined as follows:

(18) $$\begin{array}{c} \begin{array}{c} X={\left[b_{1},h_{1},b_{2},h_{2},b_{3},h_{3},b_{4},h_{4},b_{5},h_{5}\right]}^{T}={\left[x_{1},x_{2},\cdots ,x_{10}\right]}^{T},\\ \text{min }f\left(X\right)\;=100\left(x_{1}x_{2}+x_{3}x_{4}+x_{5}x_{6}+x_{7}x_{8}+x_{9}x_{10}\right),\\ \text{s}.\text{t}.\left\{\begin{array}{c} g_{1}\left(x\right)=10.7143-\frac{x_{1}x_{2}^{2}}{10^{3}}\leq 0,\\ g_{2}\left(x\right)=8.5714-\frac{x_{3}x_{4}^{2}}{10^{3}}\leq 0,\\ g_{3}\left(x\right)=6.4286-\frac{x_{5}x_{6}^{2}}{10^{3}}\leq 0,\\ g_{4}\left(x\right)=4.2957-\frac{x_{7}x_{8}^{2}}{10^{3}}\leq 0,\\ g_{5}\left(x\right)=2.1428-\frac{x_{9}x_{10}^{2}}{10^{3}}\leq 0,\\ g_{6}\left(x\right)=10^{4}*\left(\frac{244}{x_{1}x_{2}^{3}}+\frac{148}{x_{3}x_{4}^{3}}+\frac{76}{x_{5}x_{6}^{3}}+\frac{28}{x_{7}x_{8}^{3}}+\frac{4}{x_{9}x_{10}^{3}}\right)-10.8611\leq 0,\\ g_{7}\left(x\right)=x_{2}-20x_{1}\leq 0,\\ g_{8}\left(x\right)=x_{4}-20x_{3}\leq 0,\\ g_{9}\left(x\right)=x_{6}-20x_{5}\leq 0,\\ g_{10}\left(x\right)=x_{8}-20x_{7}\leq 0,\\ g_{11}\left(x\right)=x_{10}-20x_{9}\leq 0,\\ 1\leq x_{i}\leq 5,\;i=1,3,5,7,9;\;30\leq x_{i}\leq 65,\;i=2,4,6,8,10. \end{array}\right. \end{array} \end{array}$$

There are 11 constraints in this problem. Among them, *g*_1_(*x*)–*g*_5_(*x*) are related to the allowable stress constraints, *g*_6_(*x*) is the constraint regarding the allowable deflection, and *g*_7_(*x*)–*g*_11_(*x*) are restrictions to the geometric shape of the cross-section. The optimization design problem in this paper is addressed by using the penalty function method given by equation (3). The parameter settings are as described in Section 4.3.

The optimization results of MOIRCGA are compared with the optimization results in reference [27, 31, 37, 42, 50, 51]. The optimization results (the best objective function values and design variable values) that are obtained with MOIRCGA and genetic algorithm (RCGA) in reference [27, 31, 37, 42, 50, 51] are listed in Table 3, and the constraints are listed in Table 4.

It is clear from Table 3 that the function value obtained with the proposed MOIRCGA is superior to that with RCGA in reference [27, 31, 37, 42, 50, 51]. At the same time, Table 4 reveals that the optimal solution obtained by the two methods satisfies all constraints.

## 6. Conclusions

The better individuals in the population are more likely to approach the global optimal solution. However, the two offsprings generated by the LX operator are symmetric with respect to the parental position, and the two offsprings are not necessarily located near the better individual of the two parents. The two offsprings generated by the SBX operator are located on the straight line connecting the two parents, and are in the vicinity of the two parents. The two offsprings generated by the Modified SBX-crossover (MSBX) operator are located on the straight line connecting the two parents, and are in the vicinity of the two parents. The distance between two offsprings and two parents can be adaptively controlled by parameter *η*, which overcomes the deficiency of adjusting distribution index *η* which SBX cannot adaptively adjust. The two offsprings generated by the HX operator are located on the straight line connecting the two parents and are in the vicinity of the parents with better fitness value. The offsprings generated by the PCX operators are not close to the center of the parents but close to the parents. The two offsprings generated by the BLX-*α* operator are located at the center of the two parents. Since the DBX operator is only able to explore 2^*n*^ − 1 possible search directions, the search directions of DBX are limited; when the dimensionalities of the variables are small, the null vector solution is likely to be generated. Currently, the search direction is randomly generated, and the search direction is not instructive. HNDDBX overcomes the shortcomings of the above crossover operators. It not only considers the optimal individuals in the population, but also ensures that the offsprings generated by the HNDDBX operator are located near the better individual among the parents of participation crossover. Therefore, the convergence speed of the MOIRCGA using the HNDDBX operator is significantly improved.

In most previous GA algorithms, as iterations increase, the same individuals are likely to appear in the population. Therefore, it is possible that the two parents of participation crossover are the same. Under these circumstances, the crossover operation does not generate new individuals, that is, the crossover operation does not work, which affects the computational efficiency of GA and the ability to explore other extremal regions. To avoid this problem, the substitution operation is added after the crossover so that there is no duplication of the same individuals in the population. This improves the computational efficiency of MOIRCGA for quick convergence to the global optimal solution.

The local and global search abilities of different mutation operators are different. Some mutation operators have strong local search ability, and some have strong global search ability. A single mutation operator is difficult to take into account both local and global search abilities. This paper proposes a combinational mutation method which include three mutation operators, that is, Normal mutation operator, Cauchy mutation operator, and Lévy mutation operator. The Normal mutation operator has strong local search ability, and the Cauchy mutation operator has strong global search ability. The Lévy mutation operator performs local search in most cases and occasionally performs global search as well, but the global search ability of the Lévy mutation operator is superior to the Cauchy mutation operator, thus helping the algorithm avoid falling into local optimum.

The computational results with sixteen examples show that MOIRCGA has better performance than the other methods in references [27, 31, 37, 42] with respect to all the measures of performance considered.

As an example application, the optimization problem of the cantilevered beam structure is formulated, and MOIRCGA in the paper and RCGA in reference [52] are used to optimize the parameters of the cantilevered beam structure. The optimization results show that the function value obtained with MOIRCGA is superior to that obtained with RCGA.

## Figures and Tables

**Algorithm 1:**
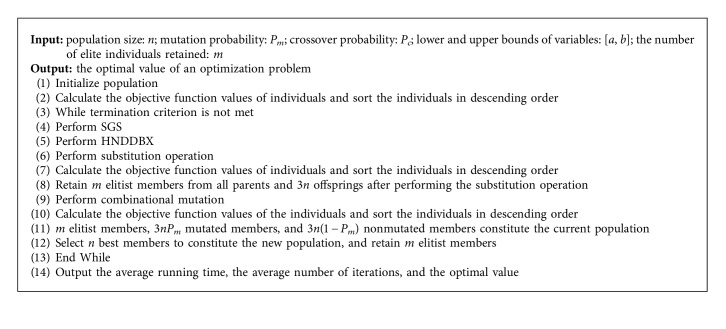
MOIRCGA.

**Table 1:** Substitution operation.

*X*	1	2	3	*X*_1_	1	2	3	*Y*	2.3	4.6	−2.6	*X*′	1	2	3
	2	5	6		2	5	6		4	3.1	−2.5		2	5	6
	3	6	7		3	6	7		−3.1	4.8	3.6		3	6	7
	1	2	3		2.5	3.1	6.3		1.5	−2.4	4.5		2.5	3.1	6.3
	2.5	3.1	6.3		7.5	−6.5	8.6						7.5	−6.5	8.6
	7.5	−6.5	8.6		3.3	6.2	−4.8						3.3	6.2	−4.8
	3.3	6.2	−4.8										2.3	4.6	−2.6
	2	5	6										4	3.1	−2.5
	7.5	−6.5	8.6										−3.1	4.8	3.6
	2	5	6										1.5	−2.4	4.5

**Table 2:** The computational results of various algorithms.

Function	IRCGA-1		IRCGA-2		IRCGA-3		SPXMOGA		HGA		MOIRCGA	
	*t*_ave_ (s)	iter_ave_ (times)	*t*_ave_ (s)	iter_ave_ (times)	*t*_ave_ (s)	iter_ave_ (times)	*t*_ave_ (s)	iter_ave_ (times)	*t*_ave_ (s)	iter_ave_ (times)	*t*_ave_ (s)	iter_ave_ (times)
*f*_1_	0.1142	89.6250	0.0709	68.1900	0.0679	51.3940	0.2024	624.2430	0.0168	17.1530	0.0155	6.2150
*f*_2_	0.0285	18.2660	0.0108	11.9562	0.0064	5.6540	0.0389	15.8500	0.0041	3.7000	0.0020	2.0330
*f*_3_	0.0842	36.2200	0.0095	13.6253	0.0120	15.7290	0.0824	20.5700	0.0068	4.8800	0.0063	3.1400
*f*_4_	0.0648	22.7620	0.0072	10.6824	0.0088	12.6810	0.1223	275.3800	0.0135	8.4500	0.0046	3.5120
*f*_5_	0.0664	26.5900	0.0186	36.2560	0.0190	38.2210	0.1317	428.9000	0.0179	23.6320	0.0158	20.8310
*f*_6_	0.0260	15.7500	0.0156	20.2610	0.0141	18.6410	0.0271	82.4800	0.0601	26.0600	0.0068	4.9460
*f*_7_	0.1305	41.1900	0.1356	242.6236	0.1287	224.1050	0.1936	263.3200	0.0862	36.6340	0.0198	22.6480
*f*_8_	0.2868	185.0700	0.2568	263.6246	0.2107	451.7110	0.6230	1785.6240	0.1962	156.2300	0.1138	127.0690
*f*_9_	0.1758	59.6400	0.0546	80.6528	0.0357	60.8970	0.1989	96.7800	0.0265	16.5600	0.0120	12.7330
*f*_10_	16.5687	11835.8600	30.6528	31042.6540	27.3205	28412.8500	35.6283	32461.6280	15.7377	10983.8614	13.2385	8414.4000
*f*_11_	0.0217	25.8100	0.0156	32.6246	0.0138	28.1210	0.1867	42.8300	0.0138	8.5320	0.0064	6.9180
*f*_12_	0.0696	50.5600	0.0653	86.6810	0.0350	56.0080	0.2302	127.5760	0.0283	21.6230	0.0194	19.5300
*f*_13_	0.1734	58.6100	0.0236	26.0625	0.0168	11.0350	0.1926	72.9426	0.0156	9.6230	0.0147	7.8780
*f*_14_	0.1431	46.7300	0.0316	65.2681	0.0287	49.7220	0.2136	98.9260	0.0289	33.2310	0.0266	31.3050
*f*_15_	0.1072	34.0400	0.1562	226.3584	0.1037	180.7190	1.8626	236.2580	0.0626	28.5300	0.0221	24.9230
*f*_16_	1.6890	1013.5681	1.7869	1926.5863	1.6216	1711.9600	6.5681	4267.8320	1.0321	1201.6560	0.8660	813.0560

**Table 3:** Optimization results of various algorithms for the cantilever beam problem.

Variables and objective function	MOIRCGA	RCGA in reference [50]	IRCGA-1	IRCGA-2	IRCGA-3	SPXMOGA	HGA
*b*_1_	3.0530	3.0459	3.005	3.0602	3.0450	3.0300	3.0442
*h*_1_	60.9997	60.8969	60.004	61.2010	60.8763	59.6231	60.8812
*b*_2_	2.8062	2.8018	3.0051	2.8160	2.8023	3.2011	2.8022
*h*_2_	56.1227	56.0168	55.10	56.3020	56.0430	55.2301	56.0432
*b*_3_	2.5236	2.5251	2.601	2.6020	2.5253	2.5891	2.5253
*h*_3_	50.4718	50.4643	50.10	50.6970	50.5041	50.0620	50.5041
*b*_4_	2.2063	2.2252	2.31	2.2237	2.2210	2.3001	2.2210
*h*_4_	44.1253	44.4745	45.46	44.2758	44.4170	45.5310	44.4167
*b*_5_	1.7498	1.7678	1.79	1.7513	1.7500	1.7921	1.7499
*h*_5_	34.9948	34.8462	35.004	35.0131	34.9950	34.5863	34.9949
*f*(*x*)	62968.18	63044.17	64387.29	63752.19	62984.70	65377.86	62980.39

**Table 4:** The constrained values of various algorithms for cantilever beam problem.

Constrained values	MOIRCGA	RCGA in reference [50]	IRCGA-1	IRCGA-2	IRCGA-3	SPXMOGA	HGA
*g*_1_(*x*)	−0.6458	−0.5814	−0.1051	−0.7479	−0.5702	−0.0571	−0.5691
*g*_2_(*x*)	−0.2675	−0.2205	−0.5521	−0.3551	−0.2301	−1.1931	−0.2299
*g*_3_(*x*)	−0.0000	−0.0020	0.0999	−0.2590	−0.0126	−0.0602	−0.0126
*g*_4_(*x*)	−0.0000	−0.1158	−0.4782	−0.0635	−0.0860	−0.4726	−0.0860
*g*_5_(*x*)	−0.0000	−0.00370	−0.0505	−0.0041	−0.0003	−0.0009	−0.0002
*g*_6_(*x*)	−0.0036	−0.00074	−0.0238	−0.2136	−0.0005	−0.1487	−0.0003
*g*_7_(*x*)	−0.0603	−0.02241	−0.096	−0.0030	−0.0237	−0.9769	−0.0028
*g*_8_(*x*)	−0.0013	−0.02071	−5.002	−0.0180	−0.0030	−8.7919	−0.0008
*g*_9_(*x*)	−0.0002	−0.03804	−1.92	−1.3430	−0.0019	−1.7200	−0.0019
*g*_10_(*x*)	−0.0007	−0.03109	−0.74	−0.1982	−0.0030	−0.4710	−0.0033
*g*_11_(*x*)	−0.0012	−0.51026	−0.796	−0.0129	−0.0050	−1.2557	−0.0031

## Data Availability

The data used to support the findings of this study are available from the corresponding author upon request.

## Appendix

### A. Test Functions

The 16 test functions are as follows:

1. *f*_1_:

(A.1) $$\begin{array}{c} \begin{array}{cc} \min & f_{1}\left(x\right)=\sum_{i=1}^{20}\left[x_{i}^{2}-10\;\cos\left(2\pi x_{i}\right)+10\right],\\ \text{s}.\text{t}. & -5.12\leq x_{i}\leq 5.12,\;i=1,2,\ldots ,20. \end{array} \end{array}$$

The global optimal solution of *f*_1_ is located at *x* ^*∗*^=(0,  0,   …,  0) with *f*_1_(*x* ^*∗*^)=0.

(2) *f*_2_:

(A.2) $$\begin{array}{c} \begin{array}{cc} \max & f_{2}\left(x\right)=-x_{1}^{2}-2x_{2}^{2}+0.4\;\cos\left(3\pi x_{1}\right)+0.6\;\cos\left(4\pi x_{2}\right),\\ \text{s}.\text{t}. & -10\leq x_{1},x_{2}\leq 10. \end{array} \end{array}$$

The global optimal solution of *f*_2_ is located at *x* ^*∗*^=(0,  0) with *f*_2_(*x* ^*∗*^)=1.

(3) *f*_3_:

(A.3) $$\begin{array}{c} \begin{array}{cc} \min & f_{3}\left(x\right)=-\sin\left(x_{1}\right){\left[\sin\left(\frac{x_{1}^{2}}{\pi }\right)\right]}^{20}-\sin\left(x_{2}\right){\left[\sin\left(\frac{2x_{2}^{2}}{\pi }\right)\right]}^{20},\\ \text{s}.\text{t}. & 0\leq x_{1},x_{2}\leq \pi . \end{array} \end{array}$$

The global optimal solution of *f*_3_ is located at *x* ^*∗*^=(2.0230,  2.0230) with *f*_3_(*x* ^*∗*^)=−1.80130.

(4) *f*_4_:

(A.4) $$\begin{array}{c} \begin{array}{cc} \min & f_{4}\left(x\right)=\left(4-2.1x_{1}^{2}+\frac{x_{1}^{4}}{3}\right)x_{1}^{2}+x_{1}x_{2}+\left(4x_{2}^{2}-4\right)x_{2}^{2},\\ \text{s}.\text{t}. & -10\leq x_{1},x_{2}\leq 10. \end{array} \end{array}$$

The global optimal solution of *f*_4_ is located at *x* ^*∗*^=(−0.0898,  0.7126) and (0.0898, −0.7126) with *f*_4_(*x* ^*∗*^)=−1.031628.

(5) *f*_5_:

(A.5) $$\begin{array}{c} \begin{array}{cc} \min & f_{5}\left(x\right)=100{\left(x_{2}-x_{1}^{2}\right)}^{2}+{\left(x_{1}-1\right)}^{2},\\ \text{s}.\text{t}. & -10\leq x_{i}\leq 10,\;i=1,2. \end{array} \end{array}$$

The global optimal solution of *f*_5_ is located at *x* ^*∗*^=(1,  1) with *f*_5_(*x∗*)=0.

(6) *f*_6_:

(A.6) $$\begin{array}{c} \begin{array}{cc} \min & f_{6}\left(x\right)=\frac{\sin^{3}\left(2\pi x_{1}\right)\sin\left(2\pi x_{2}\right)}{x_{1}^{3}\left(x_{1}+x_{2}\right)},\\ \text{s}.\text{t}. & \left\{\begin{array}{c} x_{2}-x_{1}^{2}\geq 1,\\ x_{1}-{\left(x_{2}-4\right)}^{2}\geq 1,\\ -10\leq x_{1},x_{2}\leq 10. \end{array}\right. \end{array} \end{array}$$

The global optimal solution of *f*_6_ is located at *x* ^*∗*^=(1.2279723,  4.2453733) with *f*_6_(*x* ^*∗*^)=−0.095825.

(7) *f*_7_:

(A.7) $$\begin{array}{c} \begin{array}{cc} \min & f_{7}\left(x\right)={\left(x_{1}^{2}+x_{2}-11\right)}^{2}+{\left(x_{1}+x_{2}^{2}-7\right)}^{2},\\ \text{s}.\text{t}. & \left\{\begin{array}{c} {\left(x_{1}-0.05\right)}^{2}+{\left(x_{2}-2.5\right)}^{2}-4.84\leq 0,\\ 4.84-x_{1}^{2}-{\left(x_{2}-2.5\right)}^{2}\leq 0,\\ 0\leq x_{1},x_{2}\leq 6. \end{array}\right. \end{array} \end{array}$$

The global optimal solution of *f*_7_ is located at *x* ^*∗*^=(2.246826,  2.381865) with *f*_7_(*x* ^*∗*^)=13.59084.

(8) *f*_8_:

(A.8) $$\begin{array}{c} \begin{array}{cc} \min & f_{8}\left(x\right)={\left(x_{1}-10\right)}^{3}+{\left(x_{2}-20\right)}^{3},\\ \text{s}.\text{t}. & \left\{\begin{array}{c} -{\left(x_{1}-5\right)}^{2}-{\left(x_{2}-5\right)}^{2}+100\leq 0,\\ {\left(x_{1}-6\right)}^{2}+{\left(x_{2}-5\right)}^{2}-82.81\leq 0,\\ 13\leq x_{1}\leq 100,\\ 0\leq x_{2}\leq 100. \end{array}\right. \end{array} \end{array}$$

The global optimal solution of *f*_8_ is located at *x* ^*∗*^=(14.095,  0.84296) with *f*_8_(*x* ^*∗*^)=−6961.8817.

(9) *f*_9_:

(A.9) $$\begin{array}{c} \begin{array}{cc} \min & f_{9}\left(x\right)={\left(x_{1}-2\right)}^{2}+{\left(x_{2}-1\right)}^{2},\\ \text{s}.\text{t}. & \left\{\begin{array}{c} x_{1}+x_{2}-2\leq 0,\\ x_{1}^{2}-x_{2}+2\leq 0,\\ -5\leq x_{1},x_{2}\leq 5. \end{array}\right. \end{array} \end{array}$$

The global optimal solution of *f*_9_ is located at *x* ^*∗*^=(0,  2) with *f*_9_(*x* ^*∗*^)=5.

(10) *f*_10_:

(A.10) $$\begin{array}{c} \begin{array}{cc} \min & f_{10}\left(x\right)=5\overset{4}{\underset{i=1}{\sum }}x_{i}+5\overset{4}{\underset{i=1}{\sum }}x_{i}^{2}-\overset{13}{\underset{i=5}{\sum }}x_{i},\\ \text{s}.\text{t}. & \left\{\begin{array}{cc} 2x_{1}+2x_{2}+x_{10}+x_{11}-10\leq 0,\\ 2x_{1}+2x_{3}+x_{10}+x_{12}-10\leq 0,\\ 2x_{2}+2x_{3}+x_{11}+x_{12}-10\leq 0,\\ -8x_{1}+x_{10}\leq 0,\\ -8x_{2}+x_{11}\leq 0,\\ -8x_{3}+x_{12}\leq 0,\\ -2x_{4}-x_{5}+x_{10}\leq 0,\\ -2x_{6}-x_{7}+x_{11}\leq 0,\\ -2*x_{8}-x_{9}+x_{12}\leq 0,\\ 0\leq x_{1,2,\cdots 9,13}\leq 1, & 0\leq x_{10,11,12}\leq 100. \end{array}\right. \end{array} \end{array}$$

The global optimal solution of *f*_10_ is located at *x* ^*∗*^=(1,  1,  1,  1,  1,  1,  1,  1,  1,  3,  3,  3,  1) with *f*_10_(*x* ^*∗*^)=−15.

(11) *f*_11_:

(A.11) $$\begin{array}{c} \begin{array}{cc} \min & f_{11}\left(x\right)=x_{1}^{2}+x_{2}^{2},\\ \text{s}.\text{t}. & \left\{\begin{array}{c} 2.5-x_{1}-x_{2}\geq 0,\\ x_{2}-x_{1}^{2}\geq 2,\\ -100\leq x_{1},x_{2}\leq 100. \end{array}\right. \end{array} \end{array}$$

The global optimal solution of *f*_11_ is located at *x* ^*∗*^=(0,  2) with *f*_11_(*x* ^*∗*^)=4.

(12) *f*_12_:

(A.12) $$\begin{array}{c} \begin{array}{cc} \min & f_{12}\left(x\right)=-2x_{1}+x_{2}-x_{3},\\ \text{s}.\text{t}. & \left\{\begin{array}{c} x_{1}+x_{2}+x_{3}\leq 4,\\ 3x_{2}+x_{3}\leq 6,\\ 24-20x_{1}+9x_{2}-13x_{3}+4x_{1}^{2}-4x_{1}x_{2}+2x_{1}x_{3}+2x_{2}^{2}-2x_{2}x_{3}+2x_{3}x_{1}+2x_{3}^{2}\geq 0,\\ 0\leq x_{1},x_{2}\leq 2,\\ 0\leq x_{3}\leq 3. \end{array}\right. \end{array} \end{array}$$

The global optimal solution of *f*_12_ is located at *x* ^*∗*^=(2,  0,  0) with *f*_12_(*x* ^*∗*^)=−4.

(13) *f*_13_:

(A.13) $$\begin{array}{c} \begin{array}{cc} \min & f_{13}\left(x\right)=\sum_{i=1}^{20}x_{i}^{2},\\ \text{s}.\text{t}. & -5.12\leq x_{i}\leq 5.12,\;i=1,2,\ldots ,20. \end{array} \end{array}$$

The global optimal solution of *f*_13_ is located at *x* ^*∗*^=(0,  0,   …,  0) with *f*_13_(*x* ^*∗*^)=0.

(14) *f*_14_:

(A.14) $$\begin{array}{c} \begin{array}{cc} \min & f_{14}\left(x\right)=-x_{1}-x_{2},\\ \text{s}.\text{t}. & \left\{\begin{array}{c} x_{1}x_{2}\leq 4,\\ 0\leq x_{1}\leq 4,\\ 0\leq x_{2}\leq 8, \end{array}\right. \end{array} \end{array}$$

The global optimal solution of *f*_14_ is located at *x* ^*∗*^=(0.5,  8) with *f*_14_(*x* ^*∗*^)=−8.5.

(15) *f*_15_:

(A.15) $$\begin{array}{c} \begin{array}{cc} \min & f_{15}\left(x\right)=x_{1}+x_{2},\\ \text{s}.\text{t}. & \left\{\begin{array}{c} x_{1}^{2}+x_{2}^{2}\leq 4,\\ x_{1}^{2}+x_{2}^{2}\geq 1,\\ x_{1}-x_{2}\leq 1,\\ -x_{1}+x_{2}\leq 1,\\ -2\leq x_{1},x_{2}\leq 2. \end{array}\right. \end{array} \end{array}$$

The global optimal solution of *f*_15_ is located at *x* ^*∗*^=(−1.414215754,   − 1.414211371) with *f*_15_(*x* ^*∗*^)=−2.828427125.

(16) *f*_16_:

(A.16) $$\begin{array}{c} \begin{array}{cc} \text{min } & f_{16}\left(x\right)=5.3578547x_{3}^{2}+0.8356891x_{1}x_{5}+37.293239x_{1}-40792.141,\\ \text{s}.\text{t}. & \left\{\begin{array}{c} 85.334407+0.0056858x_{2}x_{5}+0.0006262x_{1}x_{4}-0.0022053x_{3}x_{5}-92\leq 0,\\ -85.334407-0.0056858x_{2}x_{5}-0.0006262x_{1}x_{4}+0.0022053x_{3}x_{5}\leq 0,\\ 80.51249+0.0071317x_{2}x_{5}+0.0029955x_{1}x_{2}+0.0021813x_{3}^{2}-110\leq 0,\\ -80.51249-0.0071317x_{2}x_{5}-0.0029955x_{1}x_{2}-0.0021813x_{3}^{2}+90\leq 0,\\ 9.300961+0.0047026x_{3}x_{5}+0.0012547x_{1}x_{3}+0.0019085x_{3}x_{4}-25\leq 0,\\ -9.300961-0.0047026x_{3}x_{5}-0.0012547x_{1}x_{3}-0.0019085x_{3}x_{4}+20\leq 0,\\ 78\leq x_{1}\leq 102,\\ 33\leq x_{2}\leq 45,\\ 27\leq x_{i}\leq 45,\;i=3,4,5. \end{array}\right. \end{array} \end{array}$$

The global optimal solution of *f*_16_ is located at *x* ^*∗*^=(78,  33,  29.995256025682,  45,  36.775812905788) with *f*_16_(*x* ^*∗*^)=−30665.539.
